# Denosumab mimics the natural decoy receptor osteoprotegerin by interacting with its major binding site on RANKL

**DOI:** 10.18632/oncotarget.2160

**Published:** 2014-07-03

**Authors:** Aneta Schieferdecker, Mareike Voigt, Kristoffer Riecken, Friederike Braig, Thorsten Schinke, Sonja Loges, Carsten Bokemeyer, Boris Fehse, Mascha Binder

**Affiliations:** ^1^ Department of Oncology and Hematology, BMT with section Pneumology, Hubertus Wald Tumorzentrum / UCCH, University Medical Center Hamburg-Eppendorf, Hamburg, Germany; ^2^ Research Department Cell and Gene Therapy, Department of Stem Cell Transplantation, University Medical Center Hamburg-Eppendorf, Hamburg, Germany; ^3^ Department of Osteology and Biomechanics, University Medical Center Hamburg-Eppendorf, Hamburg, Germany; ^4^ Institute for Tumor Biology, University Medical Center Hamburg-Eppendorf, Hamburg, Germany

**Keywords:** denosumab, RANK, RANKL, OPG, epitope, monoclonal antibody

## Abstract

Bone homeostasis critically relies on the RANKL-RANK-OPG axis which can be targeted by the fully human monoclonal antibody denosumab in conditions with increased bone resporption such as bone metastases. The binding site and therefore the molecular mechanism by which this antibody inhibits RANKL has not been characterized so far. Here, we used random peptide phage display library screenings to identify the denosumab epitope on RANKL. Alignments of phage derived peptide sequences with RANKL suggested that this antibody recognized a linear epitope between position T233 and Y241. Mutational analysis confirmed the core residues as critical for this interaction. The spatial localization of this epitope on a 3-dimensional model of RANKL showed that it overlapped with the major binding sites of OPG and RANK on RANKL. We conclude that denosumab inhibits RANKL by both functional and molecular mimicry of the natural decoy receptor OPG.

## INTRODUCTION

Bone health relies on homeostasis of bone formation and resorption which is critically regulated by the receptor activator of nuclear factor kB ligand (RANKL), its signaling receptor RANK and its decoy receptor osteoprotegerin (OPG) [1]. The binding of osteoblast-derived RANKL to RANK expressed by osteoclast precursors promotes osteoclast differentiation and activation [2]. OPG is secreted by osteoblasts and has a very similar architecture as the RANK ectodomain [3]. It acts as a decoy receptor by binding RANKL and thereby prevents osteoclastogenesis. The structures of the RANKL-RANK and the RANKL-OPG complexes have recently been solved by co-crystallization [3-5]. At the molecular level, soluble or membrane-anchored RANKL forms a homotrimer [6, 7] that either trimerizes the RANK receptor inducing downstream signaling [8] or interacts with three OPG monomers [3], which prevent further interaction with RANK. In osteoporosis as well as in many solid tumors with bone metastases this equilibrium of RANKL, RANK and OPG is tilted towards increased levels of RANKL leading to bone resorption, pathological fractures and pain.

Denosumab, a fully human monoclonal RANKL directed antibody, prevents RANK receptor binding thereby decreasing osteoclast induced bone resorption. It is approved for the treatment of osteoporosis in post-menopausal women [9], for the prevention of skeletal-related events in patients with bone metastases [10-12] and as a preventive measure in patients undergoing hormone-deprivation in the treatment of breast and prostate cancer [13, 14]. New potential fields of application continue to arise such as the prevention of metastatic spread to the bone [1]. Despite its widespread clinical application, the precise binding site of denosumab has remained unknown.

Here, we set out to determine the denosumab epitope on RANKL to explore its mechanism of action at the molecular level. Since OPG serves as a natural decoy receptor for RANKL, we wished to establish the spatial relationship between the antibody's epitope, the OPG binding site and the critical residues for RANK binding on RANKL.

## RESULTS

### Random peptide phage display library screenings reveal a peptide motif specifically binding to denosumab

**Figure 1:**
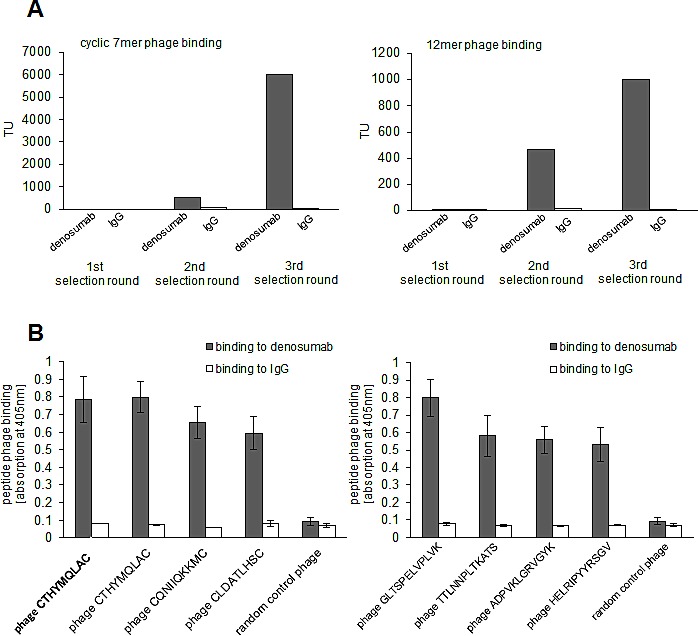
Selection of epitop mimicking phage displayed peptides on denosumab A: Denosumab binding random peptide cyclic 7mer (left panel) and 12mer (right panel) phage were enriched over three selecting rounds. IgG served as control. Enrichment was monitored by quantification of transducing units (TU) on denosumab versus control IgG recovered phage. B: Single phage clones displaying cyclic 7mer (left panel) and 12mer (right panel) peptides bind specifically to denosumab but not to control IgG. Phage binding was quantified by ELISA. Data are means from triplicates +/− SEM.

Linear 12mer and cyclic 7mer random peptide phage display libraries were screened on denosumab for epitope-mimicking peptides. Over three consecutive panning rounds, selectively binding phage were enriched (Figure 1A). Subsequently, single phage clones interacting specifically with denosumab were identified (Figure 1B). Overall, the sequenced phage clones showed a common consensus motif (Table 1). The most dominant clone CTHYMQLAC which made up 50% of sequences displayed strongest binding to denosumab and was therefore considered to mimic most reliably the presumed denosumab epitope on RANKL.

**Table 1:** Peptide sequences derived from phage display library screenings on denosumab.*

amino acid insert sequence	No. of clones
C T H R M G L A C	7
G L T S P E L P L V K	1
C L SG L R S N C	1
C Q N IL G KG C	1
C L D A T L H S C	1
T T L N N PL T K A T S	1
A D P V K L G R V GR K	1
H E L R I P YR R S G V	1
presumed epitope (233)T E R L G L G V R(241)	

* Sequences are displayed using the single letter amino acid code. A 12mer and a cyclic 7mer library were used for the screening.

### Denosumab epitope-mimicking peptides are homologous to a linear peptide strand on RANKL

We used the Mimox algorithm [15] to map the most dominant denosumab-binding peptide to the 3-dimensional surface of RANKL presuming that the peptide may mimic either a linear or conformational epitope. The software suggested a high degree of homology to a linear strand between T233 and Y241 of the protein. The presumed antibody's epitope sequence not only shared sequence homology to the most dominant epitope mimic but also to most of the remaining epitope mimics identified by our screening.

### Mutation of the presumed linear epitope abrogates binding of denosumab to RANKL

**Figure 2:**
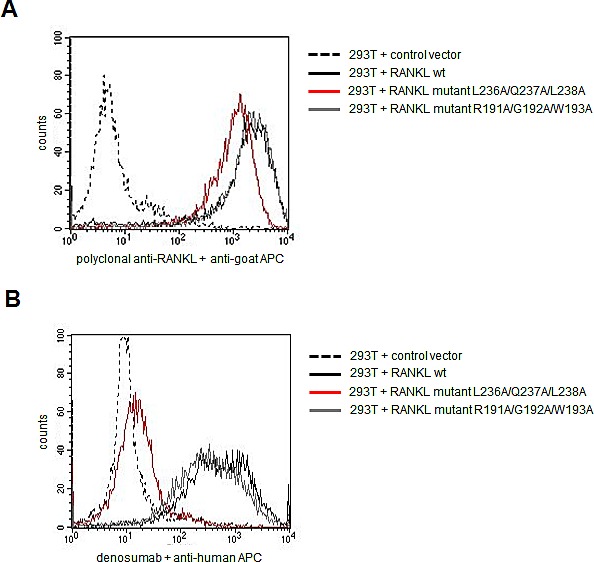
Mutational analysis confirms the presumed epitope targeted by denosumab Membrane-bound RANKL was expressed in 293T cells in either wildtype (wt) conformation or harboring mutations in core residues of the presumed epitope region (L236A/Q237A/L238A) or in a control region (R191A/G192A/W193A). Transduced 293T cells were screened for RANKL expression and denosumab binding by flow cytometry. A: Binding of polyclonal RANKL antibody to wt and mutant RANKL expressing cells. B: Binding of denosumab to wt and mutant RANKL expressing cells.

Next, we set out to definitively confirm the presumed epitope region by mutational analysis. Therefore, we cloned and expressed the wt RANKL protein in its membrane-bound form in eukaryotic 293T cells by lentiviral transduction. Both a polyclonal RANKL antibody and denosumab bound selectively to the transfected 293T cells (Figure 2). The core amino acid positions L336/Q237/L238 of the presumed epitope were then mutated to alanine by site-directed mutagenesis using the primers displayed in Supplementary Table 1. While a polyclonal RANKL directed antibody still recognized mutant RANKL, suggesting that the protein was displayed correctly on the cell surface, binding of denosumab to mutant RANKL was completely abrogated (Figure 2). In the RANKL control mutant (R191A/G192A/W193A), denosumab binding was preserved (Figure 2). This mutational analysis substantiated our hypothesis that (233)TEYLQLMVY(241) is the epitope targeted by denosumab.

### Critical amino acid residues of the denosumab epitope overlap with the major binding site II of OPG on RANKL

**Figure 3:**
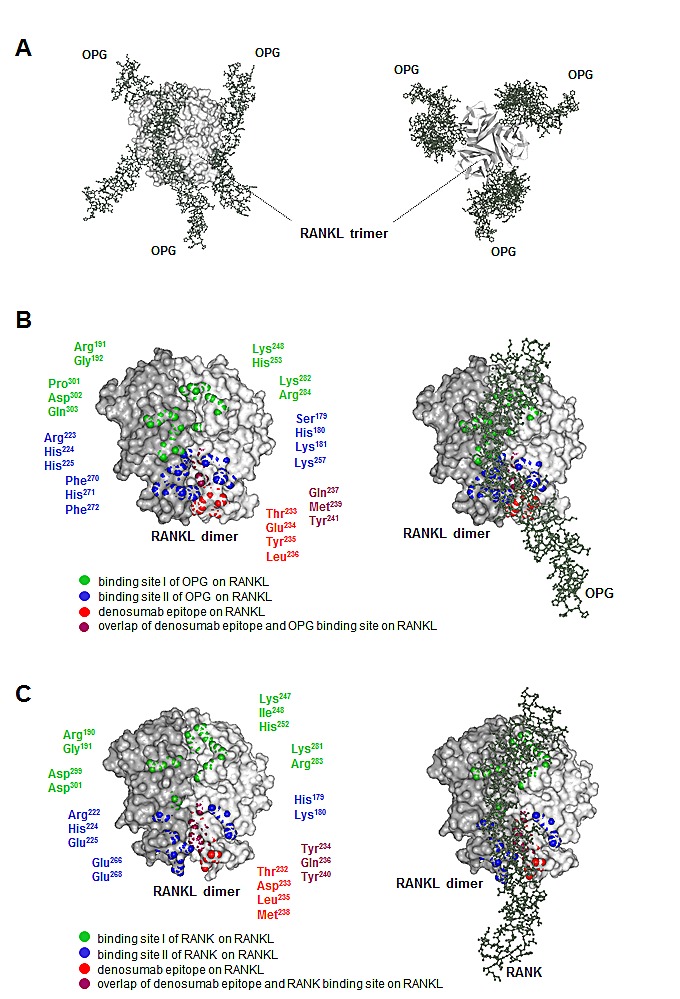
3-dimensional model of the RANK-RANKL-OPG-denosumab interaction A: Lateral view (left) and bottom-up view (right) of the human RANKL trimer (light grey) in complex with three OPG monomers (dark grey). This model was adapted from Luan et al. [pdb 3URF] [3]. B: Dimer of human RANKL (left) and its complex with OPG (right). Amino acid residues of OPG binding site I (green) and binding site II (blue) on RANKL are displayed. Model and residues were adapted from Luan et al. [pdb 3URF] [3]. In red, the denosumab epitope is shown. C: Dimer of murine RANKL (left) and its complex with murine RANK (right). Amino acid residues of RANK binding site I (green) and binding site II (blue) on RANKL are displayed. Model and residues were adapted from Nelson et al. [pdb 4GIQ] [4]. In red, the denosumab epitope is shown.

Since RANKL has been crystallized in complex with OPG (structural model shown in Figure 3A) and the two binding sites of this natural antagonist of RANKL are known, we wished to explore the spatial relationship between the denosumab epitope and the OPG binding site on RANKL. We therefore displayed both OPG interaction sites on a 3-dimensional model of RANKL (Figure 3B). Interestingly, we found that the denosumab epitope overlapped with the major binding site (binding site II) of OPG on RANKL which corresponds to the major binding site of the RANK receptor on RANKL (Figure 3C). This suggested that denosumab mimics the natural decoy receptor OPG at the molecular level and thereby prevents RANK binding and subsequent bone loss.

## DISCUSSION

The RANKL-RANK-OPG molecular triad critically regulates bone homeostasis and is therefore considered an attractive target for the treatment of osteopenic conditions. While a number of potential targeting approaches are under investigation (RANK-Fc, OPG-Fc, OPG peptidomimetics, RANKL vaccination) [16], the monoclonal RANKL inhibiting antibody denosumab is currently the most established targeted agent and has been approved for the treatment of osteoporosis and bone metastases. Moreover, denosumab prevents osteopenia in patients undergoing hormone-deprivation therapy in breast and prostate cancer and potential new fields of application are currently under investigation.

Here, we analyzed the so far unexplored molecular interaction between denosumab and RANKL by using random peptide phage display library screenings. This is an elegant technical tool to recover epitope-mimicking peptide sequences, which may be used to deduce the exact binding site of a given antibody within the parental antigen. This technology is particularly useful in identifying linear epitopes, but may also be used to explore discontinuous, structural epitopes if complemented with structural mapping software tools [15, 17, 18]. Alignment of our phage display derived peptide motif with RANKL suggested that the antibody targets a short linear epitope which could be confirmed by binding studies of mutant RANKL. Interestingly, we found that the epitope overlaps with critical amino acids within the previously characterized binding sites of OPG and RANK on RANKL. Importantly, denosumab targets amino acid residues located in one of the two OPG binding sites previously identified by co-cristallization [3]. This binding site II has been found to be especially critical for OPG binding and largely overlaps with the RANK-RANKL interaction site underlining the significance of this region for therapeutic targeting.

In addition to complementing our knowledge about ligand-receptor-antibody interactions, our data may be seen in the context of novel (preventive) targeting strategies for osteopenic conditions. Since the currently established passive targeting with RANKL directed antibody warrants continuous antibody application, direct immunization using immunogenic peptides which mimic RANKL peptide strands involved in RANK recognition could be an attractive alternative treatment strategy. Our peptide CTHYMQLAC may be suitable for this purpose since it mimics the denosumab epitope proven to be appropriate for therapeutic targeting. Such approaches, however, have to bear in mind potential pitfalls such as the natural tolerance of the immune system towards self proteins and the pronounced immunosuppression in patients with advanced cancer possibly impairing efficient humoral immune responses.

Taken together, these data reveal the structural basis for the inhibition of RANKL by denosumab and show that this therapeutic antibody functionally and molecularly mimics the naturally occuring RANKL antagonist OPG.

## MATERIALS AND METHODS

### Random peptide phage display screening

The cyclic 7mer and linear 12mer libraries were purchased from New England Biolabs. Screenings on denosumab (Xgeva^®^, Amgen) were performed after two-fold negative selection on polyclonal IgG (Intratect^®^, Biotest) essentially as previously described [19-21]. Random clones were amplified after three selection rounds and tested for selective binding to denosumab by Enzyme-linked immunosorbent assay (ELISA) as suggested by the manufacturer. Selectively binding phage were sequenced (Seqlab). Phage displaying the random peptide YMTPPLSSQQKS were used as control.

### Mapping of epitope mimics with the 3-dimensional structure of RANKL

The MIMOX algorithm was used to align the most dominant phage displayed peptide with accessible amino acids on the 3-dimensional structure of RANKL [15]. The algorithm is freely available as a web-based tool (http://immunet.cn/mimox/).

### Cloning of membrane-bound RANKL into lentiviral vector

Human RANKL cDNA (codon optimized for eukaryotic expression) was purchased from Invitrogen, digested with NotI (NEB) and EcoRI (NEB) and cloned into the third-generation self-inactivating HIV-1 derived lentiviral vector LeGO-iG3, a derivative of LeGO-iG2 [20]. The integrity of the fragment in the final vector was verified by sequencing. LeGO-G2 encoding only enhanced green fluorescent protein (eGFP) served as control.

### Mutagenesis of RANKL constructs

RANKL mutant constructs were generated using the QuikChange XL Site-Directed Mutagenesis Kit (Agilent Technologies) as described [21] using individually designed oligonucleotides (Suppl. Table 1). Successful introduction of point mutations was verified by sequencing (Seqlab).

### Transduction of 293T cells

Viral particles were produced as cell-free supernatants by transient transfection of 293T packaging cells as described [22]. To generate cell lines stably expressing the human RANKL wild-type (wt) or RANKL mutants, 293T cells (ATCC^®^ CRL-3216) were plated at a density of 5×10^4^ in 500 μl medium and transduced with the corresponding lentiviral vector. The lentiviral vectors LeGO-iG3-RANKL-wt, LeGO-iG3-RANKL-mutant and the control vector LeGO-G2 were added to the cells at equal multiplicity of infection to ensure comparable transduction rates of about 60 to 80%, as determined by FACS analysis. Transduced cells were maintained in DMEM medium containing 10% fetal bovine serum and 1% penicillin/streptomycin.

### Flow cytometry analysis

For the flow cytometry analysis, 6×10^6^ cells were incubated with 10 μg polyclonal goat anti-RANKL antibody (hTRANCE, RD Systems) or 5 μg denosumab (Xgeva^®^, Amgen). Secondary detection was performed with an anti goat-IgG APC antibody (RD Systems) or an anti human-IgG APC antibody (BD Biosciences), respectively. Cells were analyzed on a FACS Calibur (BD Biosciences). Data analysis was performed using BD CellQuest^TM^Pro software (BD Biosciences, Version 5.2.1).

### 3-dimensional models of the RANK-RANKL-OPG-denosumab interaction

The model of the human RANKL-OPG complex was adapted from Luan et al. [3], the model of the murine RANKL-RANK complex from Nelson et al. [4]. Pdb-files were downloaded from http://www.ncbi.nlm.nih.gov/structure. All illustrations were created with the ViewerLite software (Accelrys^®^, Version 4.2).

## SUPPLEMENTAL MATERIAL AND TABLE


