# Supplementary material for: Denosumab mimics the natural decoy receptor osteoprotegerin by interacting with its major binding site on RANKL
Source: Oncotarget. 2014 Jul 3;5(16):6647–53. doi: 10.18632/oncotarget.2160 (PMC4196153; doi:10.18632/oncotarget.2160)
Supplement: Supplementary file 1 [file oncotarget-05-6647-s001.pdf]

Denosumab mimics the natural decoy receptor osteoprotegerin by interacting with its major binding site on RANKL

Supplementary Material

Supplementary Table 1: Denomination and sequences of mutagenesis primers.*

**primer denomination**	**primer sequence**
RANKL 236/237/238 for	GGCCACAGAGTACGCGGCGGCGATGGTGTACGTGACC
RANKL 236/237/238 rev	GGTCACGTACACCATCGCCGCCGCGTACTCTGTGGCC
RANKL 191/192/193 for	GCTGGTATCACGACGCGGCCGCGGCCAAGATCAGC
RANKL 191/192/193 rev	GCTGATCTTGGCCGCGGCCGCGTCGTGATACCAGC

*All primers are designed in a 5’-3’ orientation. Base exchanges are underlined.
